# Statistical-Shape Prediction of Lower Limb Kinematics During Cycling, Squatting, Lunging, and Stepping—Are Bone Geometry Predictors Helpful?

**DOI:** 10.3389/fbioe.2021.696360

**Published:** 2021-07-12

**Authors:** Joris De Roeck, Kate Duquesne, Jan Van Houcke, Emmanuel A. Audenaert

**Affiliations:** ^1^Department of Human Structure and Repair, Ghent University, Ghent, Belgium; ^2^Department of Orthopedic Surgery and Traumatology, Ghent University Hospital, Ghent, Belgium; ^3^Department of Trauma and Orthopedics, Addenbrooke’s Hospital, Cambridge University Hospitals NHS Foundation Trust, Cambridge, United Kingdom; ^4^Department of Electromechanics, Op3Mech Research Group, University of Antwerp, Antwerp, Belgium

**Keywords:** lower limb kinematics, bone geometry, musculoskeletal modeling, statistical shape model, SSM-based kinematics, model optimization

## Abstract

**Purpose:** Statistical shape methods have proven to be useful tools in providing statistical predications of several clinical and biomechanical features as to analyze and describe the possible link with them. In the present study, we aimed to explore and quantify the relationship between biometric features derived from imaging data and model-derived kinematics.

**Methods:** Fifty-seven healthy males were gathered under strict exclusion criteria to ensure a sample representative of normal physiological conditions. MRI-based bone geometry was established and subject-specific musculoskeletal simulations in the Anybody Modeling System enabled us to derive personalized kinematics. Kinematic and shape findings were parameterized using principal component analysis. Partial least squares regression and canonical correlation analysis were then performed with the goal of predicting motion and exploring the possible association, respectively, with the given bone geometry. The relationship of hip flexion, abduction, and rotation, knee flexion, and ankle flexion with a subset of biometric features (age, length, and weight) was also investigated.

**Results:** In the statistical kinematic models, mean accuracy errors ranged from 1.60° (race cycling) up to 3.10° (lunge). When imposing averaged kinematic waveforms, the reconstruction errors varied between 4.59° (step up) and 6.61° (lunge). A weak, yet clinical irrelevant, correlation between the modes describing bone geometry and kinematics was observed. Partial least square regression led to a minimal error reduction up to 0.42° compared to imposing gender-specific reference curves. The relationship between motion and the subject characteristics was even less pronounced with an error reduction up to 0.21°.

**Conclusion:** The contribution of bone shape to model-derived joint kinematics appears to be relatively small and lack in clinical relevance.

## Introduction

Differences in motion patterns can be attributed to a large number of associated variables: velocity, proprioceptive, vestibular, and visual stimuli as well as neurocognitive and executive functions, body weight, sex, aging effects, and pathological deviations (Schwartz et al., 2004; Chau et al., 2005; Martin et al., 2013; Kobayashi et al., 2016; Reznick et al., 2020). While intuitively vital, the impact of bone and joint geometry on *in vivo* motor variability, nonetheless, remains controversial (Hoshino et al., 2012; Freedman and Sheehan, 2013; Lynch et al., 2020). Recent work on the knee, however, tends to indicate a significant relation between the joint anatomy and both experimentally and model-derived joint motions (Smoger et al., 2015; Nesbitt et al., 2018; Clouthier et al., 2019). Whether these findings can be extrapolated to a possible accurate statistical prediction of multi-body kinematics from mainly bone geometry predictors, remains to be investigated.

Recent advances in computational methodology allow for improved characterization of bone morphometry as well as motion at a population wide level. Statistical shape modeling enables to describe individualized bone geometry more precisely than consensus bone geometry or linearly scaled generic bone models (Audenaert et al., 2019a; Cerveri et al., 2020; Nolte et al., 2020). Similarly, statistical modeling of kinematics by non-linear methods as well as improvements in curve alignment methods during the pre-processing phase, might provide more reliable and stronger correlations between human anatomy and motion as opposed to previous reports (Freedman and Sheehan, 2013; Moissenet et al., 2019; De Roeck et al., 2020). Nevertheless, acquiring perfect kinematic data in an ethically responsible way remains a sticking point. *In vivo* kinematic data can be acquired by means of bone pins, radio-stereometric-analysis or fluoroscopy. However, these invasive and radiation exposing methods in healthy participants are cumbersome because of ethical concerns, and additionally they interfere with normal anatomy and physiological processes (Matsuki et al., 2017; Galvin et al., 2018). In contrast, skin-mounted marker motion capturing does not cause any associated hazards and therefore is the standard for healthy cohorts to date. However, the accuracy of marker-based or optoelectronic motion capture systems is affected by soft tissue artifacts (Andersen et al., 2009; Leardini et al., 2017; Begon et al., 2018; Galvin et al., 2018; Niu et al., 2018; Van Houcke et al., 2019; De Roeck et al., 2020). Several approaches have been developed to deal with these errors, such as multibody kinematics optimization methods (Lu and O’Connor, 1999; Andersen et al., 2009; Leardini et al., 2017; Begon et al., 2018) or by combining the motion tracking system with ultrasound (Niu et al., 2018).

Understanding how bone morphometry affects joint function might reveal fundamental insights into how geometrical features contribute to musculoskeletal disorders (Clouthier et al., 2019). However, the actual relationship between these two entities, kinematics and anatomical shape, remains largely unanswered in literature and low predictability of kinematics based on geometry characteristics has been reported (Lynch et al., 2020).

The objectives of this paper are therefore twofold. First, we intend to quantify individual differences for a wide range of activities of daily living (ADL) at a population-wide level. Statistical kinematic models that aim to describe the inter-subject variance in natural joint motion are appropriate for this purpose (Chau et al., 2005; Deluzio and Astephen, 2007; Leardini et al., 2017; Reznick et al., 2020; Duquesne et al., 2021). Secondly, we aspire to improve upon the understanding the extent to which segmented bone morphometry or subject characteristics are related to model-derived lower limb kinematics.

## Materials and Methods

### Sample Recruitment

A group of able-bodied males aged between 18 and 25 years was recruited to establish the relationship between morphometric and motion variability. A healthy and homogeneous group was chosen to minimize potential bias from clinical (e.g., neurological and musculoskeletal pathology) origin or age-related differences. Therefore, individuals with musculoskeletal disorders or history of surgery were excluded. A second prerequisite to participate involved the absence of overweight (i.e., BMI less than 25 kg/m^2^). An overview of population demographics (*n* = 57) is provided in Table 1. The study was approved by the Ghent University Hospital Ethics Committee and informed consent was obtained from all participants. As demonstrated in previous work on lunge dynamics, a minimal sample size of 50 is required to reproduce biomechanical waveform data at a population covering level (De Roeck et al., 2020).

**TABLE 1:** General characteristics of the investigated population.

**Population descriptors**	**Mean (95% CI)**	**Standard deviation**
Age (years)	22.1 (21.5–22.7)	2.2
Length (cm)	181.4 (179.8–183.0)	6.3
Weight (kg)	71.5 (69.5–73.5)	7.8
CE angle (°)	28.2 (26.9–29.4)	4.8
Alpha angle (°)	64.5 (62.4–66.5)	8.1
CCD angle (°)	129.2 (128.0–130.3)	4.6
Femoral anteversion (°)	8.8 (6.8–10.9)	8.0

*CI, confidence interval; CE angle, center-edge angle (hip acetabulum); CCD angle, caput-collum-diaphyseal angle or femoral neck-shaft angle.*

### Bone Geometry Segmentation and Modeling

The gold standard to obtain individualized bone geometry is the segmentation of shapes from high resolution 3D medical imaging (Nolte et al., 2020). Therefore, the pelvis and lower limb bones of the study cohort were scanned using a 3-Tesla MAGNETOM Trio-Tim System MRI device (Siemens AG, Erlangen, Germany). Following, segmentation procedures were applied to extract the underlying bone geometry. Automatic, model-based segmentation and registration was performed using the Ghent lower limb model (Audenaert et al., 2019b). For the construction of this model, a total of 606 (left + right side) medical images were previously acquired and analyzed. We refer to the former work for detailed information on model construct and validation (Audenaert et al., 2019a, 2020). Shape model accuracy root-mean-square errors (RMSE) amounts 0.59 ± 0.08 mm, 0.59 ± 0.06 mm, and 0.59 ± 0.06 mm, for the pelvis, femur, and shank bones, respectively. To represent 95% of shape variance, the number of required shape modes in the pelvis, femur, and shank model was estimated at 33, 7, and 6 components, respectively (Audenaert et al., 2019b). By doing so, the combined bone geometry training set was assumed to be accurately described and compactly parameterized, at a population-covering level (Audenaert et al., 2019a). All of these modes were significant in the rank of roots permutation test developed by Vieira (2012). A combined shape model including femur (thighbone), tibia (shinbone), and fibula (calf bone) was additionally defined, with the aim to describe the entire lower limb for the canonical correlation analysis. All analyses were conducted in MATLAB (R2020b, Mathworks, Natick, MA, United States).

### Motion Analysis

Twenty-seven skin beads (12 mm) were applied to the bony landmarks of the pelvis-leg apparatus and one at the vertebra prominens. Markers were placed by the same investigator and according to standardized protocols (Gorton et al., 2009; De Roeck et al., 2020). This is crucial as improper marker positioning can induce variability in the kinematics, particularly in the offsets between the kinematic curves of different subjects (Gorton et al., 2009). Spatial marker trajectories were measured using the marker-based 8-camera optical motion capture system from Optitrack (Natural Point Inc., Corvallis, OR, United States).

Subjects were asked to perform several ADL activities. More specifically, the motion analysis included stationary cycling, squat, lunge, and stair movements. Before motion tracking was initiated, each test subject received a brief training of the intended movements. The purpose of this instruction moment was to secure an adequate and smooth recording of the motor tasks. Then each movement was executed and recorded twice. All experiments were conducted in the same setting and under the same circumstances to limit the influence of external factors.

#### City Bike

**FIGURE 1:**
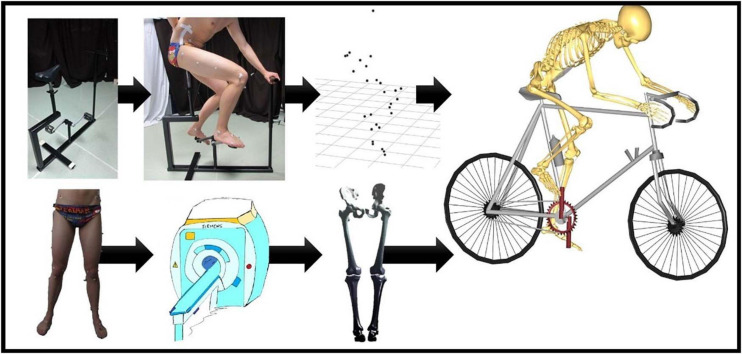
Flow chart of the musculoskeletal simulation in the Anybody Modeling System. On the upper left, there is the bike model that was used by our test panel during the cycling experiments. Motion was recorded frame by frame through skin marker registration. Each subject underwent MRI for determination of the skin tags. Subsequently, Anybody calculated the kinematics for all the recorded frames. An identical workflow was applied for the simulation of squat, lunge, and stair movements.

To mimic the bicycle movements, a bike model was constructed. As provided in Figure 1, the bike model consisted of two pedals, a saddle, and steering handles. For standardization purposes, the test subjects received clear instructions on how to position themselves on the bike and changing the height of the steering handles and the saddle. Subsequently, the volunteers were asked to complete at least three full pedaling cycles.

#### Race Bike

Subjects were asked to take a seat on the bike model while maximally bending over, to mimic the posture of a professional cyclist. The height of the saddle was adjusted to be equal to the height of the hips of the subject, standing next to the model.

#### Squat

Squatting is one of the most challenging motions for the hip and knee joints as it generates considerably high reaction forces and it approximates the fully functional flexion range of the lower limb (Schellenberg et al., 2018; Van Houcke et al., 2019). Each subject slowly bent his legs while keeping his heels on the floor. Thereupon, the subjects were asked to hold the resulting position for 2 s, after which they returned to their original starting position.

#### Lunge

Like the squat, lunging is a closed-chain movement. The subjects stepped approximately 0.6–1.0 m forward with the right leg onto the other force plate. Consequently, both knees bent at the same time. Ultimately, the subjects stood still in this position for a few seconds and pushed off the right foot to rise. The recording ended when the starting position was reached.

#### Stair Movements

Subjects stood on a step and smoothly stepped up or down to the second step. As for the lunge, the volunteers were asked to use their right leg first. Both ascent and descent staircase motions were modeled individually. Given a previous study showed knee peak flexion angles to be correlated with the step height, the stair level was fixed (Niu et al., 2018).

#### Musculoskeletal Simulation Analysis

To simulate the ADL activities, the segmented bones, the position of the pelvic, thigh, and shank markers, the motion capture trajectories and force plate data were imported into the AMS (version 7.1.0). For each subject, individualized musculoskeletal models were created using the anybody managed model repository (AMMR) (version 2.0) and the Twente lower extremity model (TLEM) 2.0 (Carbone et al., 2015). Standard simplified joint definitions of the generic human body model were utilized, which include a 3 degree of freedom ball-and-socket joint for the hip joint, a 1 degree of freedom hinge joint for the knee joint, and a 2 degree of freedom condylar joint at the ankle (i.e., flexion and eversion). For the geometrical personalization of the lower limb, we employed a previously developed automated workflow (Van Houcke et al., 2019). Herein, first landmark correspondence between the individual bone geometry and the Anybody template bone geometry is established using the non-rigid registration algorithm of Audenaert et al. (2019b) and Audenaert (2021). Subsequently, automatic non-linear scaling of the musculoskeletal geometry based on the individualized bone geometries of the pelvis, right thigh, and right shank was performed. The left thigh and shank were assumed to be symmetric and were reconstructed through mirroring the corresponding right sided bones. For kinematic analysis, the position of the pelvic, thigh and shank reflective markers relative to the bone was directly imported in contrast to the normal workflow where the marker positions are estimated and optimized. As the positions of the skin markers are one of the factors highly influencing the joint kinematics, importing the positions eliminates one of these (El Habachi et al., 2015). Furthermore, to minimize skin shift effect an overdetermined kinematic solver tracking the experimental markers in a least-squares sense was used (Andersen et al., 2009).

#### Registration of Lower Limb Kinematics

We evaluated the hip flexion, hip abduction, hip rotation, knee flexion, and ankle flexion of the right leg in a single model for each movement. First, the simulation output from AMS was trimmed based on the knee flexion angles. As such, simulation output denoting subject immobility was rejected (Van Houcke et al., 2019; De Roeck et al., 2020). Then, each kinematic curve was discretized into 101 registration entries assigning 0–100% of movement progression (Sadeghi et al., 2003; Schwartz et al., 2004; Chau et al., 2005; Deluzio and Astephen, 2007; Kobayashi et al., 2016; Matsuki et al., 2017; Bouças et al., 2019; Moissenet et al., 2019; Van Houcke et al., 2019; De Roeck et al., 2020; Reznick et al., 2020; Duquesne et al., 2021; Warmenhoven et al., 2021). At last, a continuous registration (CR) method was applied to remove the phase variability of the curves (Sadeghi et al., 2003; Chau et al., 2005; Duquesne et al., 2021). CR is an alignment technique which converts the unaligned curves into perfectly aligned curves using a warping function. Curves are said to be perfectly aligned with a template curves if they only differ in amplitude. In an iterative process, CR tries to find a warping function that aligns the functional approximation of the waveforms perfectly with the estimated sample mean curve (the template curve). The process is repeated until the estimated sample mean (new template) does not differ significantly from the previously obtained estimated sample mean (the template curve). As such, the curve registration approach contributes to a reduction of the inter-subject variability (Sadeghi et al., 2003). Especially peak values and pronounced features in gait curves will be influenced after implementing the registration (Sadeghi et al., 2003; Duquesne et al., 2021). Regarding the cycling registrations, which curves imply a periodic nature, a Fourier basis was used as a functional approximation of the curves. For the curves of other movements, a spline basis was implemented to fit the kinematic data.

#### Parameterization of Lower Limb Kinematics

Once the pre-processing was completed, the registrations were parameterized. Therefore, all data was mean centered to examine the variability. To extract the leading dimensions in the kinematic curves, principal component analysis (PCA) of waveforms was applied. PCA of waveforms has been widely used in the literature for the modeling of gait curves (Chau et al., 2005; Deluzio and Astephen, 2007; Kobayashi et al., 2016; Warmenhoven et al., 2021). For each movement, a parameterized model was created based on covariance-based PCA. PCA decomposes the kinematics *K* into independent principal components (PC), corresponding to the eigenvector *P* of the covariance matrix ${\left(K-\bar{K}\right)}^{T}\left(K-\bar{K}\right)$, as outlined by Jolliffe (1986). $\bar{K}$ serves as the average kinematic curve and *b* equals to the vector of PC weights or modes.

$$K=\bar{K}+Pb$$

The number of significant PC was derived by means of the rank of roots algorithm (Vieira, 2012). Model compactness and accuracy were calculated for each of the motion tasks. The compactness refers to the number of principal components involved in the model. Further, accuracies from our training dataset *K* consisting of *n* samples and 101 time frames *t* were presented by a RMSE.

$$RMSE=\frac{1}{101n}\sum_{i=1}^{n}\sum_{t=1}^{101}{∥K_{model,i}\left(t\right)-K_{i}\left(t\right)∥}^{2}$$

### Correlation and Regression Analysis

Canonical correlation (CCA) and partial least squares regression (PLSR) are highly related to each other. However, the emphasis is slightly different. In CCA, the aim is to maximize correlation and to allow for a statistical interpretation of this correlation. CCA is a useful tool to understand the relationship between multiple explanatory variables and a set of response variables (Thompson, 2005). In contrast, PLSR maximizes the covariance, and is typically done for predictive purposes. Therefore, in this work CCA was used to report on the statistical correlation between the motion and shape modes, while PLSR was used to define the predictive value of bone shape.

Given the profound dominance of size in statistical shape models (Audenaert et al., 2019a), CCA was applied on the first PC weight vector from the kinematic model and the PC weights of the shape samples. Additionally, the correlation between a set of general demographic characteristics (i.e., age, length, and weight) of our test subjects and the main kinematic mode was established, similar to the gait prediction studies of Bouças et al. (2019) and Moissenet et al. (2019). Correlations were tested for significance by means of the Wilks’ lambda likelihood ratio statistic (α = 0.05). The null hypothesis states there is no correlation.

Partial least squares regression was used to predict the kinematic modes starting from the shape modes or the subject characteristics. To minimize overfitting of the data, only one partial least square regression component was used. To assess the regression fit, reconstruction errors of the shape-specific kinematic predictions were benchmarked again the RMSE when imposing the average curve for all subjects. Again, differences were tested by means of the two-tailed pairwise *t*-test (α = 0.05).

$$RMSE=\frac{1}{101n}\sum_{i=1}^{n}\sum_{t=1}^{101}{∥K_{prediction,i}\left(t\right)-K_{i}\left(t\right)∥}^{2}$$

## Results

**FIGURE 2:**
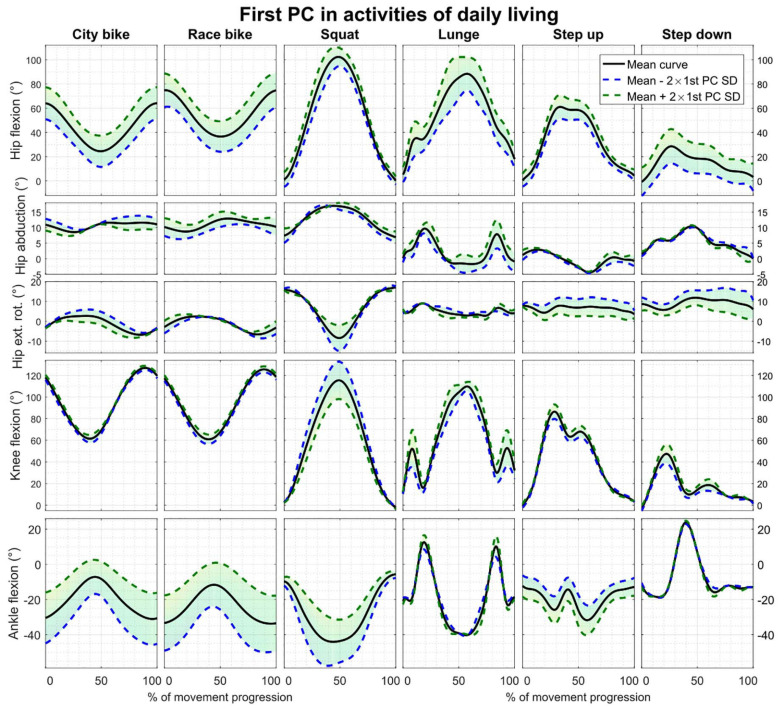
First principal component of all the parameterized kinematic models based on PCA. Model training curves were first aligned by means of CR. The black line depicts the average motion curve, while the green and blue dashed line represent 2 standard deviations (SD) of the first kinematic mode.

Reconstruction errors of the statistical kinematic models vary between 1.09° and 3.54° and are listed in Table 2. The city bike, race bike, and step up models all consist of five principal components retaining 92.43, 93.91, and 79.64% of population variance, respectively. Conversely, the squat, lunge, and step down models are composed of seven principal components having 88.22, 78.87, and 85.33% of population variance, respectively. The interpretation of the main principal component from the six kinematic models is outlined in Figure 2.

**TABLE 2:** Root-mean-square errors (RMSE ± standard deviation) from the kinematic parameterization.

**RMSE (°)**	**Hip flexion**	**Hip abduction**	**Hip external rotation**	**Knee flexion**	**Ankle flexion**
City bike	1.09 ± 0.07	1.56 ± 0.08	1.62 ± 0.08	1.44 ± 0.09	2.09 ± 0.13
Race bike	1.12 ± 0.08	1.51 ± 0.10	1.78 ± 0.10	1.22 ± 0.08	1.82 ± 0.12
Squat	2.58 ± 0.18	1.53 ± 0.08	2.19 ± 0.13	2.21 ± 0.15	1.80 ± 0.09
Lunge	2.82 ± 0.11	2.48 ± 0.12	2.77 ± 0.13	3.54 ± 0.17	3.33 ± 0.14
Step up	1.79 ± 0.09	1.91 ± 0.08	1.87 ± 0.10	2.12 ± 0.16	2.38 ± 0.10
Step down	1.82 ± 0.09	1.77 ± 0.08	1.93 ± 0.09	2.07 ± 0.09	2.16 ± 0.08

**FIGURE 3:**
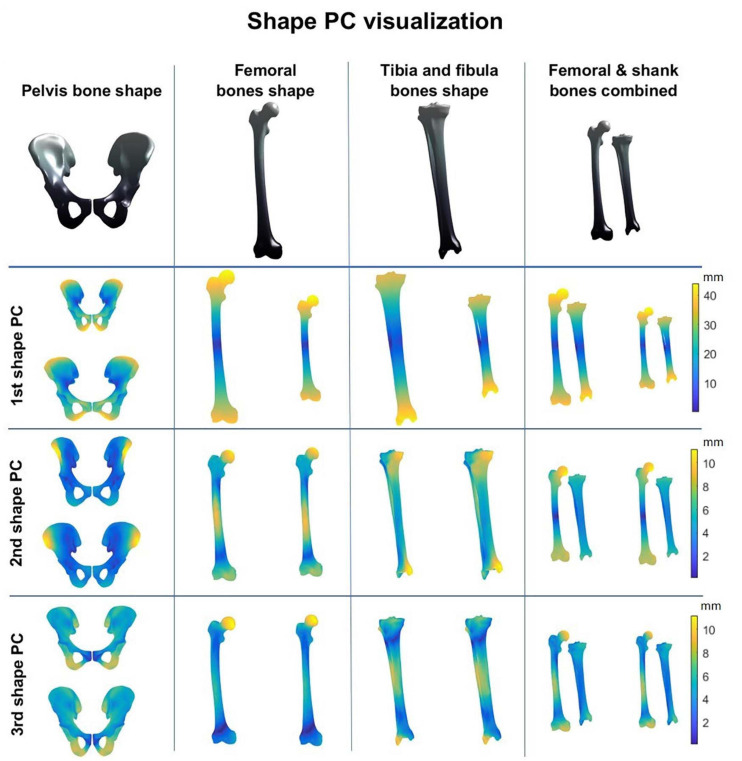
Shape modes from the personalized bone shape models of pelvis, femur, shank (tibia + fibula), and femur and shank combined. Averaged geometry is displayed at the top while variation is represented with a color scheme (average shape ± 3 standard deviations of the shape principal components).

A combined shape model was introduced involving the femur, tibia and fibula bone geometry. Herein, the first 10 modes reproduce 95% of shape variance in the data and all of them are significant according to the rank of roots permutation test. The 3 dominant principal components from the 4 geometry models are shown in Figure 3. Furthermore, the results of the canonical correlation analyses are summarized in Table 3. Overall, canonical correlation coefficients are weak and the correlations between the kinematic model and the pelvis geometry seem of interest, yet likely attributable to overfitting, considering the large amount of shape components required to describe pelvic anatomy.

**TABLE 3:** Canonical correlation analysis between the significant shape PC weights or biometric variables and the first kinematic mode.

**Kinematic model**	**Demographics/anatomy**				
	Age, length, and weight	Pelvis bone shape	Femoral bone shape	Tibia and fibula bones shape	Femur and shank bones combined
Correlation measure	r^2^ (p)	r^2^ (p)	r^2^ (p)	r^2^ (p)	r^2^ (p)
City bike	0.1479 (p = 0.036)	0.7012 (p = 0.060)	0.2420 (p = 0.249)	0.2170 (p = 0.355)	0.3578 (p = 0.245)
Race bike	0.0911 (p = 0.178)	0.6758 (p = 0.157)	0.2401 (p = 0.291)	0.2248 (p = 0.357)	0.4127 (p = 0.128)
Squat	0.0433 (p = 0.543)	0.7399 (p = 0.078)	0.2769 (p = 0.210)	0.1502 (p = 0.779)	0.4156 (p = 0.174)
Lunge	0.2559 (p = 0.002)	0.7787 (p = 0.009)	0.3364 (p = 0.053)	0.1731 (p = 0.619)	0.4902 (p = 0.026)
Step up	0.0643 (p = 0.331)	0.5881 (p = 0.498)	0.1873 (p = 0.544)	0.2570 (p = 0.227)	0.4555 (p = 0.057)
Step down	0.1129 (p = 0.093)	0.6210 (p = 0.276)	0.1202 (p = 0.850)	0.1795 (p = 0.549)	0.2943 (p = 0.509)
					

Finally, the performance of the PLS regression is presented in Table 4. The RMSE on the predicted curves are all significantly lower compared to reference curve (i.e., average motion curves) benchmarking for each ADL. However, these corrections were modest and clinically insignificant, ranging from 0.14° to 0.41° when using bone shape as regression input and between 0.04° and 0.11° when subject characteristics serve as input variables. As such, only 1.4% (step down) up to 16.6% (race cycling) of kinematic variance could be explained by statistical shape models. On the other hand, merely 1.4% (step up) up to 7.3% (lunge) of kinematic variance could be explained by age, length, and weight.

**TABLE 4:** Partial least squares regression of the demographics and the combined femur and shank model PC weights to predict ADL kinematics.

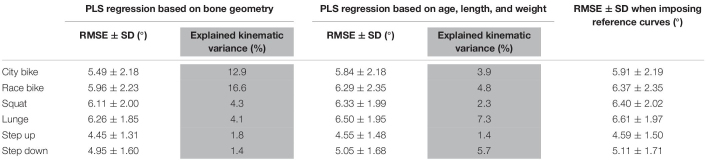

*Predictions are benchmarked again the RMSE when imposing the average curve. All RMSE differences were significant at the 0.05 level. The percentages of explained kinematic variance by the regression models are also given. SD, standard deviation.*

## Discussion

The canonical correlations between the shape modes and kinematic modes are weak, even for the first mode which is predominantly representing the overall size. The association between the set of subject characteristics and kinematics is also found to be weak with still less explained kinematic variance, lower correlation coefficients and even less prediction ability. As such, our findings are similar to the trial from Moissenet et al. (2019) in which lower limb sagittal kinematics (hip, knee, and ankle flexions) during gait were predicted based on demographic parameters (walking speed, gender, age, and BMI) with errors exceeding 5°. In summary, when considering healthy Caucasian males aged between 20 and 25 years, correlation and predictions between shape and kinematic modes were not found to be clinically relevant. Hence, it seems that the applied modeling and statistical approaches are overkill methodology to the problem. In contrast, it appears that the statistical predictions based on some basic demographic parameters (i.e., without predictors based on bone geometry) might be as valid for the prediction of hip flexion, abduction, and rotation, knee flexion, and ankle flexion similar as reported in the literature for gait (Rasmussen et al., 2020).

While the presented methodology could not demonstrate important shape related variability in motion patterns, future work is mandatory to investigate such in pathological mixed groups, where the impact of bone geometry abnormalities is likely to be of higher importance. The presented methodology seems adequate to investigate these patterns.

The results of our research are affected by some limitations. Although further investigations might extend our findings, our study cohort represents a very selected group, especially with minimal age and height differences among subjects, and accordingly their kinematic variability was limited (Kobayashi et al., 2016). Furthermore, the sexual dimorphism of pelvic, femoral and tibial bone morphometry were not taken into account in the correlation analysis, since our study involved male subjects only (Audenaert et al., 2019a). While this approach minimized the impact of confounding variables and allowed us to deploy a well-controlled methodological pipeline, it limits the extent to which our conclusions can be extrapolated. Findings might be different in pathological conditions, with possibly more sources of variability and eventually with more meaningful and notable patterns. Clearly, more work in this area is needed.

Secondly, our findings are specific to the five model-derived joint angles approach used and the way the bone geometry is taken into account in the multibody kinematics optimization process. As such the study design aimed for the detection of obvious and large scale kinematic features such as walking with toes pointed outward. Our findings can therefore not be entirely generalized. For example, subtle relationships have been previously reported in the literature between joint shape and 6 degrees of freedom tibio-femoral kinematics (Smoger et al., 2015; Valente et al., 2015; Nesbitt et al., 2018; Clouthier et al., 2019; Martelli et al., 2020).

Furthermore, this study relies on the assumption that subject-specific motion can be predicted by a small set of parameters. Reconstruction errors of the statistical kinematic models generally range around 2 degrees, which corresponds to the inter-session error in the gait study from Schwartz et al. (2004) on lower-limb kinematics. Further, these errors are in line with the measurement errors classically found in optoelectronic experiments in a review from Leardini et al. (2017) comparing marker-based registrations to fluoroscopy and bone pins experiments.

Lastly, the sparse amount of data remains a major drawback in our investigation. Therefore, the regression analysis should be interpreted cautiously, and one must be aware of the potential risk of overfitting. Moreover, the pelvis shape model is notably less compact than the other models and therefore less suitable to regression analysis, particularly when having restricted numbers of training samples. Thereupon, pelvis bone morphometry was not incorporated into our combined shape model. Even though intra-subject variability was minimized by CR, no obvious patterns could be found here to link between bone morphometry and observable patterns in motion tasks. Alternatively, prediction performance may improve using deep learning methodology, however, such would require sample sizes to be substantially forced up (Bouças et al., 2019).

In conclusion, motion curves are not prominently related to subject characteristics or personal bone geometry in the present study. Furthermore, when benchmarked against average kinematic reference curves, personalization based on bone geometry appears to lack in clinical relevance.

## Data Availability Statement

The datasets generated for this study are available on reasonable request to the corresponding author.

## Ethics Statement

The studies involving human participants were reviewed and approved by Ghent University Hospital Ethics Committee. The patients/participants provided their written informed consent to participate in this study.

## Author Contributions

JD, KD, and EA designed the algorithms. JD, JV, and EA assisted in the data collection and manipulation. JD and KD carried out the statistical analysis. JD wrote the first draft of the manuscript. All authors contributed to the manuscript revision and approved the submitted version.

## Conflict of Interest

The authors declare that the research was conducted in the absence of any commercial or financial relationships that could be construed as a potential conflict of interest.
